# Genome Editing and Improvement of Abiotic Stress Tolerance in Crop Plants

**DOI:** 10.3390/life13071456

**Published:** 2023-06-27

**Authors:** Rakesh Kumar Yadav, Manoj Kumar Tripathi, Sushma Tiwari, Niraj Tripathi, Ruchi Asati, Shailja Chauhan, Prakash Narayan Tiwari, Devendra K. Payasi

**Affiliations:** 1 Department of Genetics & Plant Breeding, College of Agriculture, Rajmata Vijayaraje Scindia Krishi Vishwa Vidyalaya, Gwalior 474002, India; rakeshyadav07081996@gmail.com (R.K.Y.); sushma2540@gmail.com (S.T.); ruchiasati.95@gmail.com (R.A.); chauhanshailu24@gmail.com (S.C.); 2 Department of Plant Molecular Biology & Biotechnology, College of Agriculture, Rajmata Vijayaraje Scindia Krishi Vishwa Vidyalaya, Gwalior 474002, India; tiwarisprakashn051194@gmail.com; 3 Directorate of Research Services, Jawaharlal Nehru Krishi Vishwa Vidyalaya, Jabalpur 482004, India; 4 Regional Agricultural Research Station, Sagar 470001, India; dpayasi@gmail.com

**Keywords:** abiotic and biotic stress, CRISPR, mega nucleases, TALEN, ZFN

## Abstract

Genome editing aims to revolutionise plant breeding and could assist in safeguarding the global food supply. The inclusion of a 12–40 bp recognition site makes mega nucleases the first tools utilized for genome editing and first generation gene-editing tools. Zinc finger nucleases (ZFNs) are the second gene-editing technique, and because they create double-stranded breaks, they are more dependable and effective. ZFNs were the original designed nuclease-based approach of genome editing. The Cys2-His2 zinc finger domain’s discovery made this technique possible. Clustered regularly interspaced short palindromic repeats (CRISPR) are utilized to improve genetics, boost biomass production, increase nutrient usage efficiency, and develop disease resistance. Plant genomes can be effectively modified using genome-editing technologies to enhance characteristics without introducing foreign DNA into the genome. Next-generation plant breeding will soon be defined by these exact breeding methods. There is abroad promise that genome-edited crops will be essential in the years to come for improving the sustainability and climate-change resilience of food systems. This method also has great potential for enhancing crops’ resistance to various abiotic stressors. In this review paper, we summarize the most recent findings about the mechanism of abiotic stress response in crop plants and the use of the CRISPR/Cas mediated gene-editing systems to improve tolerance to stresses including drought, salinity, cold, heat, and heavy metals.

## 1. Introduction

By the end of the year 2050, the world population is anticipated to reach up to 10 billion [1]. In this situation, increasing food crop production by 60% over the coming decades is necessary to ensure global food security [1,2]. To sustainably increased food production, additional integration of all developed relevant techniques, such as genomics, genome editing (GE), artificial intelligence, and deep learning, will be necessary [3,4]. Crop modification methods have a long history and have been used ever since the first agricultural plants were domesticated. Since then, other new methods have been created and are being developed to boost crop production and economic value even more. Traditional crop breeding techniques in the 20th century either relied on naturally occurring mutations or on mutagenesis that was created artificially [5]. Genetic research has traditionally focused on the identification and assessment of spontaneous mutations. Scientists were reliant on each other and showed that radiation or chemical treatment could increase the rate of mutagenesis [6,7]. Later approaches, suchas radiation and chemical mutagenesis, altered the genome at random sites by inserting transposon motifs that may be induced in some animals. However, a fundamental disadvantage of conventional breeding methods is the length of time needed to breed new varieties of any crops with the required agronomic characteristics. The duration of the growing season and the maturity level of the plants (particularly long-period growers, such as trees), as well as various stages of crossing, selection, and testing during the breeding process, all have an impact on this [8]. The plant genome cannot be targeted using conventional techniques for chemical and physical mutagenesis or natural mutations. Using genetic engineering, better plants and animals may be developed more quickly [5].

The first genetically modified (GM) crops were released for sale in 1996 [9]. Generations of GM crops up to now have relied on the genome’s random insertion of new DNA sequences. The possibility that the inserted gene may affect or impede the activity of other crucial nearby genes has been raised as a concern regarding this approach. In addition, public anxiety regarding GM crops is increased when talking about the introduction of ‘alien’ genes from distantly related organisms, which is thought to be ‘unnatural’ despite mounting evidence to the contrary [10,11].

The creation and use of DNA-based markers at the turn of the twenty-first century has made it possible to reduce significantly the time needed to generate new lines and varieties of agricultural crops [10,11,12,13]. All these factors have greatly helped the development of focused GE methods [14,15,16,17]. In yeast and mice, the first targeted genetic alterations were created in the 1970s and 1980s [6,8]. This gene targeting was based on the homologous recombination process, which was extremely accurate.

RNA interference (RNAi) was one of the first GE technologies [5,18,19]. Even though this technology has been successfully used in functional genomics and plant breeding [20,21,22], it has several drawbacks, including the unlimited insertion site of an RNAi construction into the genome and partial gene function suppression [5].

This is a marvelous time for genetics, due to advances in genetic analysis and genetic manipulation. Genome editing, the most recent crop-enhancement method, allows precise changes of the plant genome by deleting undesired genes or enabling genes to acquire new functions [23]. Numerous crops’ genomes have been sequenced, and improvements in genome-editing techniques have made it possible to breed for desired features. To sustainably increase food production, additional integration of all developed relevant techniques, such as genomics, genome editing (GE), artificial intelligence, and deep learning, is necessary [24].

Advanced biotechnological methods are made possible by genome-editing tools, allowing for precise and effective targeted modification of an organism’s genome. Several novel tools for genome or gene editing are available to enable researchers to modify genomic sequences precisely [25]. These techniques facilitate novel insights into the functional genomics of an organism and enable us to alter the regulation of gene expression patterns in a pre-determined region. Because of accurate DNA manipulation, genome-editing technologies, for instance, CRISPR/Cas9 (clustered regularly interspaced short palindromic repeats/CRISPR-associated systems), TALENs (transcription activator-like effector nucleases), CRISPR/Cas12a (Cpf1, CRISPR from *Prevotella* and *Francisella*1), and Cas9-derived DNA base editors, provide unprecedented advancements in genome engineering. As a result, this technology is a powerful tool that can be employed to secure the global food supply [26].

Genome editing was first proposed by Capecchi [27] in the 1980s. This method allows for the removal, modification, or addition of genetic material at specified genomic locations. Even though current GE technologies are substantially more accurate than traditional mutagenesis [28,29], the biggest barrier here is still the legitimacy of GE crops. Assessing the biosafety of such crops is a unique difficulty because it is impossible to predict the effects of single base alterations following the application of ODM and BEs [30,31].

**Figure 1:**
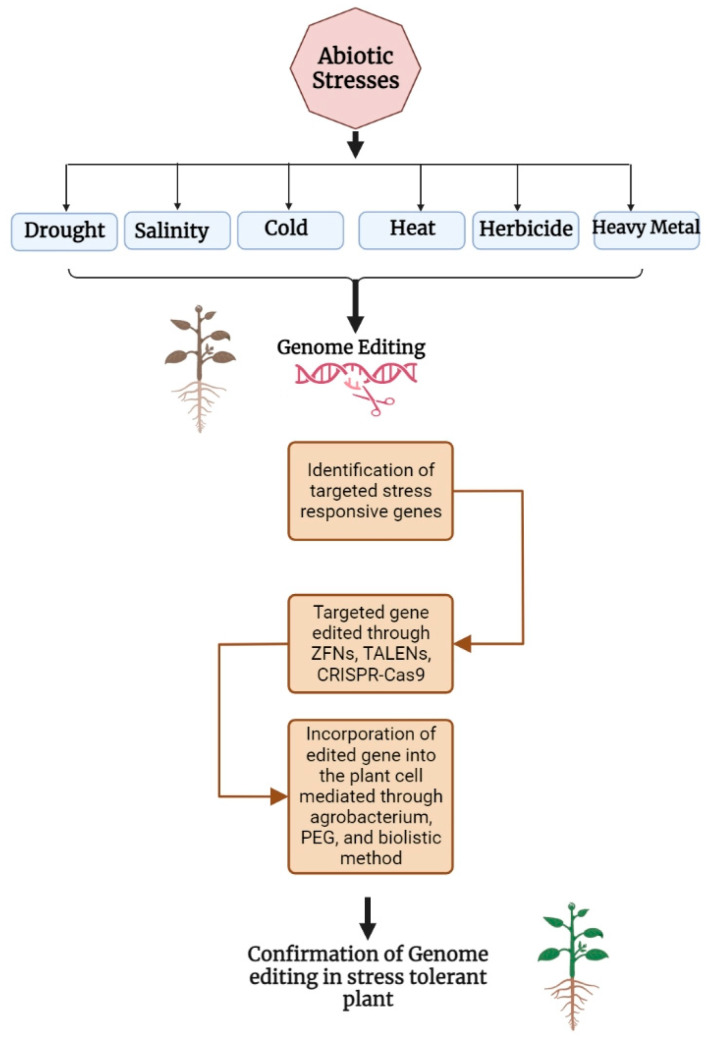
Applications of genome editing in crop improvement against abiotic stresses.

The primary elements that affect plant growth and reduce agricultural productivity are biotic stressors [32,33] such as disease and insect pests, along with abiotic stressors [13] including cold, drought, and saline–alkali stress (Figure 1). Many crop plants that can withstand abiotic stress have previously been created via traditional marker-assisted breeding. However, due to extensive screening [34,35] and backcrossing procedures, it takes this tactic about a decade to generate abiotic stress-resilient crops effectively [36]. Although genetically modified, stress-tolerant plants have disclosed encouraging results, several barriers still stand in the way of their widespread commercialization. In many ways, crops with genome editing differ from genetically engineered species [37]. Considering this, genome editing seems to be a sophisticated strategy to create crops that are resistant to different abiotic stress in the future, because it allows precise manipulation of different gene loci in comparably less time, which lowers the cost of crop-improvement programmes [38]. Gene-editing technology based on CRISPR/Cas might successfully target complex quantitative genes linked either directly or indirectly to abiotic stressors. The use of CRISPR-Castechnology has been linked in recent years to the establishment of disease resistance in plants by modifying gene regulation [39,40,41,42]. Currently, CRISPR/Cas-based genome editing has been efficaciously utilized to investigate tolerance against multiple abiotic stresses, including heat, drought, salt, and nutritional values in several critical agricultural plants [43,44]. In this review article, we summarize the most likely uses of the CRISPR/Cas9-mediated genome editing technique in crop plants for dealing with diverse abiotic stresses such as heat, drought, salinity, cold, herbicide etc., and we predict the tools for future advancements in the creation of crop varieties that can withstand stresses.

## 2. Genome-Editing Strategy

Genome editing is one of the most promising approaches to understand the genome and to improve crop plants. The fundamental mechanisms involved in genetic modification by programmable nucleases (NHEJ) are the recognition of target genomic loci and binding of effector DNA-binding domain (DBD), double-stranded breaks (DSBs) in target DNA caused by restriction endonucleases (FokI and Cas), and repair of DSBs through homology-directed recombination (HDR) or non-homologous end joining [45]. While the well-organized and error-prone NHEJ results in the deletion or insertion of nucleotides, the less efficient and more accurate HDR results in the replacement of nucleotides. Genome-editing methods such as ZFN, TALEN, and CRISPR/Cas are being utilized to add the desired trait(s) and remove the undesirable ones. Numerous techniques are available for genome editing using either a site-specific recombinase (SSR) system or a site-specific nuclease (SSN) system. Both systems must be able to find a known sequence. The SSN system causes single or double strand DNA breaks and activates endogenous DNA repair systems. Depending on how the sites (loxP, FLP, etc.) are oriented, SSR technology, such as Cre/loxP- and Flp/FRT-mediated systems, can knockdown or knock in genes in the eukaryotic genome around the area of the target [46].

Plant genome-editing techniques have been classified into four major types based on onsite-specific endonucleases (Table 1). Those are ZFNs, meganucleases, TALENs, and CRISPR-Cas9 along with DSB-free genome editing, base editing, prime editing, and mobile CRISPR. These techniques are all discussed in detail below.

### 2.1. Zinc-Finger Nucleases

ZFNs are assemblages of DNA recognition modules based on zinc fingers and the DNA cleavage domain of the FokI restriction enzyme. With their use, the target genome can be altered to introduce a variety of genetic changes, such as deletions, insertions, inversions, translocations, and point mutations [47]. They have two domains, the first of which is a nuclease domain and the second of which is a DNA-binding domain. The DNA-binding domain’s 3- to 6-zinc finger repeats may recognize nucleotide sequences that are 9 to 18 bases long. The second domain is made up of the restriction enzyme Flavobacterium okeanokoites I (FokI), which is necessary for DNA cleavage [48].This method involves three artificial restriction enzymes, specifically ZFN-1, ZFN-2, and ZFN-3 [49]. ZFN-1: At this point, ZFN is transferred to the plant genome devoid of taking a repair template. Once it arrives at the plant genome, it makes double-stranded breaks (DSB) to the host DNA leading to non-homologous end joining (NHEJ) of DNA [50], which either produces site-specific arbitrary mutations or a small deletion or insertion. ZFN-2: Distinct from ZFN-I, a homology-directed repair (HDR) alongside a short repair template is delivered to the crop genome next to the ZFN enzyme [51]. The template DNA is homologous to the target DNA, which attaches to a specific sequence causing a double-stranded rupture. The template commences repairing with an endogenous repair mechanism which is directed to site-specific point mutations throughout homologous recombination (HR). ZFN-3: As soon as the ZFN transcribing gene is transferred to the plant genome next to the large repair template, it is called ZFN3 [51,52].

ZFN has been effectively implemented in *Arabidopsis*, tobacco, soybean, and maize [53,54,55,56]. In one example of the use of ZFNs in crop breeding, the insertion of PAT gene cassettes disrupted the endogenous ZmIPK1 gene in maize, which altered the inositol phosphate profile of growing maize seeds and improved herbicide resistance [53].ZFNs can be created utilizing various protein-engineering techniques to target essentially any unique DNA stretch [57]. ZFNs with enhanced specificity and activity have been developed to produce knockouts, which disable the gene’s function, as well as gain-of-function alterations [58].

### 2.2. Meganucleases

Longer DNA sequences (more than 12 bp) can be selectively detected and cut by meganucleases, which are endonucleases. This approach has been discovered in a wide variety of organisms, including archaebacteria, bacteria, algae, fungi, yeast, and many plant species. Meganucleases at the target region can sustain mild polymorphisms [59]. Meganucleases have been divided into five groups based on their sequence and structural features. These consist of His-Cys box, GIY-YIG, LAGLIDADG, PD-(D/E) XK, and HNH [60,61].Genome editing has mostly used members of the LAGLIDADG meganuclease (LMN) family. According to Silvaet al. [60], the name of this protein family is taken from the sequence of the main motif found in its structure. LMNs are typically expressed in the chloroplast and mitochondria of unicellular eukaryotes. The bulk of these endonucleases are dimeric proteins that have two separate functions: they splice their own introns as RNA maturases and cleave exon sequences as specialized endonucleases [62]. I-SceI and I-CreI’s genomes can be edited employing the rRNA gene of the mitochondrial DNA of *Saccharomyces cerevisiae*. The 21S contains the I-SceI gene’s location. The chloroplast of *Chlamydomonas reinhardtii*, a unicellular alga, was found to contain I-CreI, which is found in the 23S rRNA gene. However, due to the difficulties in reengineering meganucleases to target specific DNA areas, their utility in genome editing is limited [63].

### 2.3. Transcription Activator-like Effector Nucleases (TALENs)

Restriction enzymes called TALENs, or transcription activator-like effector nucleases, are designed to cleave specific DNA sequences. TALENs are made up of a nuclease that can cleave DNA in cells and a TALE domain that is intended to mimic the natural transcription activator-like effector proteins. Currently, a huge number of researchers are studying transcription activator-like effector nucleases (TALENs), which are composed of a free designable DNA-binding domain and a nuclease [64], in a variety of organisms. TALENs have recently emerged as a cutting-edge method for genome editing in a variety of species and cell types. It was discovered that TALENs may alter the genome in a variety of plants, including *Arabidopsis*, Nicotiana, Brachypodium, barley, potatoes, tomatoes, sugarcane, flax, rapeseed, soybean, rice, maize, and wheat [65,66]. According to a report, rice was the first crop in which TALENs technology was employed for enhancement. According to Li et al. [67], the main pathogen of blight disease (*Xanthomonas oryzae*) significantly reduces global rice production each year. By disrupting the genes for fatty acid desaturase (FAD), soybeans with high oleic acid and low linoleic acid levels were produced, improving the shelf life and heat stability of soybean oil [68,69]. TALENs are naturally occurring type III effector proteins created by *Xanthomonas species* that change the host plant’s gene expression. The TALENs proteins comprise a nuclear localization signal, a transcriptional activation domain, and a core DNA-binding domain [70]. The nuclear localization signal helps TALENs enter the nucleus, whilst the activation domain activates the transcriptional machinery to start expressing genes [71].

### 2.4. Clustered Regularly Interspaced Short Palindromic Repeats (CRISPR)/CRISPR-Associated Protein 9 (Cas9)

**Figure 2:**
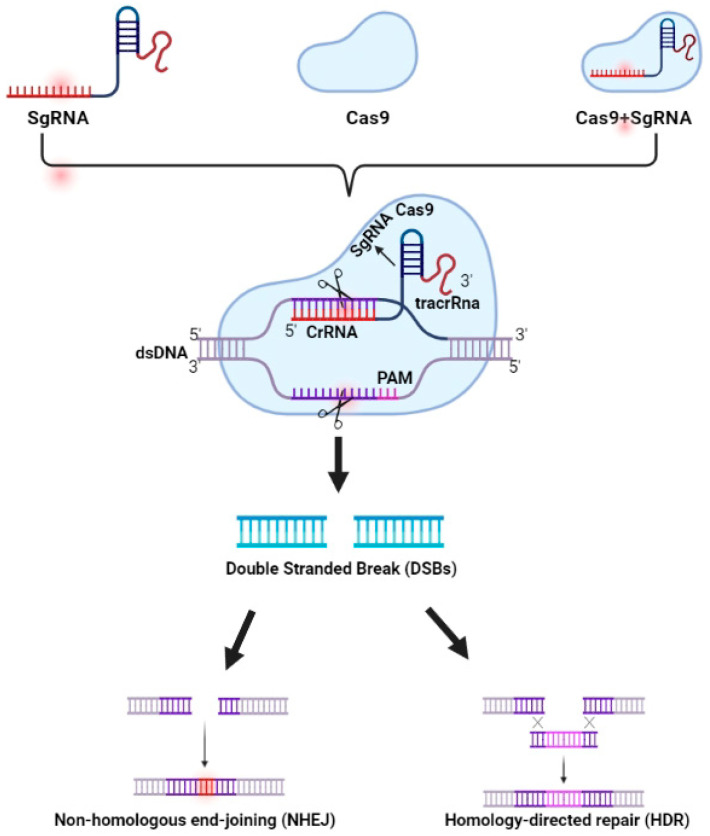
Mechanism of genome editing using CRISPR/Cas9.

Clustered regularly interspaced short palindromic repeats (CRISPR/Cas9) are short, repetitive genetic variations that are present in most bacterial and archaeal species. CRISPR/Cas9 and its associated proteins produce a very strong defensive system that works as a safeguard for plants against foreign agents including bacteria, viruses, and other elements. The first application of CRISPR/Cas9 in an adaptive immune system was documented in a 2007 experiment [72]. The CRISPR/Cas9 gene-editing system has revolutionized research in animal and plant biology since its usage in genome editing was first demonstrated in mammalian cells in 2012 [73]. According to Rathore et al. [23] first-generation CRISPR/Cas9 genome editing involves simple manipulationand cloning techniques that can be applied to a variety of guide RNAs to edit different locations in the targeted organism’s genome (Figure 2). With the use of CRISPR/Cas, crop species can be precisely edited, opening the door to the generation of favorable germplasm and new, more sustainable agricultural systems. The genetic modification of crops can now be targeted and precise due to recent developments in CRISPR/Cas9 technology, hastening the advancement of agriculture [42]. To date, only a few species have been studied using this methodology [74].The yield, quality, disease resistance, and climatic adaptability of monocots and dicots have all been improved by the CRISPR/Cas9 system [75]. The genomes of cereal crops including wheat, maize, rice, and cotton as well as fruits and vegetables such as tomatoes and potatoes have all been altered using the CRISPR/Cas9 technique [76,77].

According to Makarova et al. [78], the CRISPR/Cas system can be divided into three types: type I, type II, and type III. Bacteria and archaea both have type I CRISPR/Cas mechanisms based on the exact signature of the Cas protein. The Cas3 protein’s endonuclease activity is used to connect to the DNA sequence [78]. In bacteria, the type II CRISPR/Cas system has been developed. The four protein pairs Cas1, Cas2, Cas4/Csn2 proteins, coupled with Cas9, make up the simplest system. The type III CRISPR/Cas system hunts for DNA and RNA in archaea, as well as infrequently in bacteria. Cas6, Cas10, and repeat associated mysterious proteins (RAMP) are markers for its presence. Cas10 protein’s processing of crRNA ultimately aims to cleave DNA [78]. The *Streptococcus pyogenes* (SpCas9)-derived type II CRISPR system mostly targets the negatively regulating genes [79].

The CRISPR/Cas technique is straightforward, stable, and enables effective change compared withthe first two generations of genome-editing systems. These traits allowed CRISPR/Cas to quickly replace the traditional genome-editing methods ZFN and TALEN. The techniquewas adapted from the bacterial defense mechanism. The CRISPR/Cas mechanism is used by a variety of bacterial and archaeal species to protect themselves against invading viruses [80]. Many studies are now being conducted to improve the CRISPR/Cas system and increase the tool’s ability to target the genome. For instance, non-canonical NGA and NG PAM sites in plants may be found using xCas9, SpCas9-VRQR, and Cas9-NG variants [81,82]. SpCas9 orthologues have been recognized from *Streptococcus thermophiles* (St1Cas9), *Staphylococcus aureus* (SaCas9), *Streptococcus canis* (ScCas9), and *Brevibacillus laterosporus* (BlatCas9).They have been demonstrated to amend plant genomic loci with PAM sequences of NNGRRT, NNG, NNAG AAW, and NNNCND, respectively [83,84]. Additionally, the type V Cas12a and Cas12b extracted from different bacterialsystems have been demonstrated with AT-rich PAM specifications and employed in genome editing of selected plants [85,86].

The CRISPR/Cas9 gene-editing approach has so far been used on more than 20 crop species to increase yields and reduce biotic and abiotic stress [87]. Genome-editing techniques based on CRISPR/Cas9 have been utilized to enhance agricultural disease resistance and tolerance to severe abiotic environments including salinity and drought. Three rice genes involved in regulating responses to various abiotic stress stimuli, including phytoene desaturase (OsPDS), betaine aldehyde dehydrogenase (OsBADH2), and mitogen-activated protein kinase (OsMPK2), have undergone sequence-specific CRISPR/Cas9-mediated genomic modification. CRISPR/Cas9 technology was successfully used by Shan et al. [88] to insert the TaMLO gene (mildew resistance locus O) into wheat protoplasts. It was also discovered that *Blumeria graminis* f. sp. Tritici, the agent of powdery mildew illness, is resistant to the CRISPR TaMLO knockdown (Btg). Wheat ethylene responsive factor3 (TaERF3) and wheat dehydration response element binding protein 2 (TaDREB2) are two abiotic stress-related genes that were targeted by the CRISPR/Cas9 genome-editing technology in wheat protoplasts, according to Kim et al. [89]. The CRISPR/Cas9 technology can be used in conjunction with current and upcoming breeding techniques such as speed breeding and omics-assisted breeding to boost agricultural production and ensure food security (Table 2).

**Table 1:** Comparison of different types of plant genome-editing techniques.

Feature	ZFNs	Meganucleases	TALENs	CRISPR/Cas	References
Length of target sequence (bp)	18–36 bp	12–40 bp	28–40 bp	20–22 bp	[90,91]
Nuclease protein	FokI	I-SceI	FokI	Cas9 proteins	[91,92,93]
Dimerization	Required	Not required	Not required	Not required	[90,91,92]
Mode of action	Double-stranded break in target DNA	Direct conversions in targeted regions	Double-stranded break in target DNA	Double-stranded breaks or single-stranded nicks in target DNA	[94,95,96]
Repair events	NHEJ	HDR	HDR	NHEJ	[92,93,97]
Mutagenesis	High	Middle	Middle	Lower	[94]
Cloning	Necessary	Not necessary	Necessary	Not necessary	[91,98,99]
Creation of libraries and multiplexing	Challenging	Challenging	Challenging	Possible	[91,96,99]
Cost	Higher	Higher	Higher	Low	[100]
Types	One	One	One	Many	[101]
Specificity	Moderate	High	High	Low	[90,91]
Crop improvement	Low	Low	Low	High	[100]
Future use	Medium	Medium	Medium	High	[100]

**Table 2:** List of reported targeted gene(s) via ZFNs, TALEN, and MNs gene-editing tool technologies in different plant species to develop resistant/tolerant genotypes.

Crop	Gene	Trait	Technique	References
Rice	*OsQQR*	Detection of safe harbor loci herbicide	ZFNs	[102]
	*OsBADH2*, *OsDEP1*, *OsSD1*, *OsCKX2*	Fragrance	TALEN	[103]
	*Os11N3*	Bacterial blight resistance	TALEN	[67]
	*OsCSA*	Photoperiod sensitive male sterility	TALEN	[104]
	*OsDERF1*	Drought tolerance	TALEN	[104]
Wheat	*TaMLO-A1*, *TaMLO-B1*, *TaMLO-D1*	Resistance to powdery mildew	TALEN	[105]
Maize	*PAT*	Herbicide resistance	ZFNs	[106]
	*ZmIPK1*	Herbicide tolerant and phytate reduced maize	ZFNs	[53]
	*ZmTLP*	Trait stacking	ZFNs	[107]
	*ZmPDS*, *ZmIPK1A*, *ZmIPK*, *ZmMRP4*	Biosynthesis of phytic acid	TALEN	[108]
	*MS26*	Independent lines of male sterile plants	MNs	[109]
Barley	*HvPAPhy*	Phytase reduction and seed development	TALEN	[110]
Soybean	*DCL*	Herbicide transmission	ZFNs	[111]
	*FAD2-1A*, *FAD2-1B*	Low polyunsaturated fats	TALEN	[68,69]
Tobacco	*GUS: NPTII*	Chromosome breaks	ZFNs	[112]
	Endochitinase-50 gene (*CHN50*)	Emergence of resistance to herbicides	ZFNs	[113]
Tomato	*L1L4*/*NF-YB6*	Reduced contents of the anti-nutrient’s oxalic acid	ZFNs	[114]
Cotton	*EPSPS*	Herbicide tolerance	MNs	[115]
	*Hppd*	Herbicide tolerance	MNs	[115]
Potato	*VInv*	Sugar metabolism	TALEN	[116]

### 2.5. DSB-Free Genome Editing

A sole histidine residue at site 840 of the HNH domain of SpCas9 cuts the PAM strand, while the aspartate at site 10 in the RuvC domain cuts the opposite strand3. Mutating both amino acids to alanines (D10A and H840A) resulted in nuclease-dead Cas9 (dCas9). dCas9 still identifies its target site and frees up the DNA in an R-loop without including DSBs. The binding of dCas9 to its solitary target site can work as a repressor of transcription and is called CRISPR interference (CRISPRi). Alternately, dCas9 can be utilized as a tool for localization of DNA effector proteins to the genome. Examples of this approach are CRISPR–DNMT3 fusion proteins and CRISPR activators (CRISPRa) for targeted methylation. DNA-alteration enzymes are combined with dCas9 to induce genetic variants for overcoming the limitations linked with DSB initiation in genome engineering [117].

### 2.6. Base Editing

The first base editor combines dCas9 to the cytidine deaminase apolipoprotein B mRNA editing catalytic polypeptide-like (rAPOBEC1), which catalyzes the alteration from cytidine to uracil. The cell mends this uracil into thymidine, resultingin an assembly (BE1) replacing a C•G by a T•A base pair, entitled a cytosine base editor (CBE) [118]. First-generation CBEs were suppressed by uracil glycosylation. So, second-generation base editors (BE2) were invented by combining an uracil glycosylase inhibitor (UGI) with the dCas9–rAPOBEC1 combination [119].For increasing editing efficiency, dCas9 can be changed into a nickase SpCas9-D10A (BE3). The strand not altered by rAPOBEC1 is cleaved. The cell identifies this nick and starts DNA repair to solve the damage. The strand withthe base modification is used as a template for repairing the nick to yield stable integration. The BE3 architecture was furthermore ameliorated by combining an additional UGI in fusion with linker optimization to result in a fourth-generation cytosine base editor (BE4). BE4s have improved editing efficiency by approximately50%, with two-fold decline of unintended byproduct formation such as point mutations and indels [118]. Subsequent ancestral reconstitution and codon optimization led to a CBE architecture that enables the most powerful base editing in organoids, 2D cell lines, and in vivo by improving nuclear localization and expression of the proteins [120].

### 2.7. Prime Editing

The logic behind prime editing is to escort exogenous DNA with the modification of interest close to the Cas9 binding site. Areverse transcription (RT) domain obtained from the Moloney murine leukaemia virus was combinedwith nickase SpCas9- H840Atodevelopthe first generation of prime editors (PE1). The RT domain changes RNA into DNA tofind its template in the 3′ extension of the specially designed sgRNA, entitledthe primeediting guide RNA (pegRNA).Itguides the Cas9 in PE1 to the target site. After targetrecogination, the PAM-consistingstrand is nicked by the active HNH domain of Cas9-H840A. Then, the pegRNA extension combineswiththe nicked strand of the primer-binding site (PBS).Then, the RT domain of PE1 uses the restpegRNA(RT template) to synthesize a 3′-DNA flap containingthe edit of interest. This DNAflap is solved by cellular DNA repair procedure combining the edit of interest [121]. Theprime editing requires optimizing PE3guides andpegRNA, limiting its implementationin organoids. Threemodifications have been made forovercoming this issue. First, the utilizationof two pegRNAs in trans alongwith overlayingRT domains enhancesprime-editing competencein plants [121]. Second, engineered pegRNAs can have tmpknot or evopreqdomains combinedatthe 3′ end. These domains enhancethe stability of the pegRNA [122]. Finally, including the N394Kand R221K amino acid alterationincreases the nuclease workof SpCas9, resulting in a more efficient PE2Max [123].

### 2.8. Mobile CRISPR

A breakthrough in the CRISPR tool, “genetic scissors” was announced by scientists of the Max Planck Institute of Molecular Plant Physiology to edit plant genomes. The discovery could speed up and simplify development of novel and genetically stable crop varieties by fusing grafting with a ‘mobile’ CRISPR tool. The drawing of the CRISPR/Cas9 gene scissors is transferred as RNA from the rootstock of a genetically modified plant to the grafted shoot of a normal plant. The gene scissors protein is made with the aid of the RNA. This gene scissor protein edits specific genes in flowers. Plants carry the desired gene modification in the next generation. A normal shoot is grafted onto roots containing a mobile CRISPR/Cas9, which allows the genetic scissor to move from the root into the shoot. It edits the plant DNA without leaving a trace of itself in the subsequent generations of plants. This ground-breaking turn can save cost and time and evade current limitations of plant breeding.

## 3. Genome Editing Related to Abiotic Stresses

Abiotic stresses that impact plant growth and development, such as salt, drought, extremely high temperatures, cold, and heavy metals, can reduce agricultural production by approximately 50% [124].Numerous biochemical, morphological, and physiological factors important for plant development are influenced by stress. Stresses from the environment can modify how plants behave as they develop. Most changes in plant growth and development caused by different abiotic stresses are associated with poorer yields [13]. By 2050, the rapid growth in the human population is predicted to reach 9.7 billion. The global temperature is also set to increase significantly. As plant scientists, it is hard for us to manage the food requirements of the increasing population. However, we own the capability to develop climate-flexible crop varieties that can flourish under such challenging circumstances. These varieties must be maintained in ruthless climatic conditions such as heat, drought, heavy metals, cold, or flood stresses. This requires a continuous search for newer and diverse germplasm [125,126], which was traditionally performed either entirely through development of natural variations [127,128] or by selective breeding [129,130]. Another possibility is the construction of mutant populations that are evaluated to hunt for new resources among variations that might be novel valuable mutations that in turn are included in breeding programmes. Modern genome-editing system tools such as CRISPR facilitate the user to commence desirable genomic modifications accurately, illustrating great promise as a tool for producing novel climate-resistant plants [131]. In over 20 agronomically important crops, CRISPR/Cas mediated gene editing is widely utilized and accepted for crop improvement against different abiotic stresses [79].

Ordinarily, plants are equipped with numerous defense schemes against abiotic stresses. Among numerous defense mechanisms of abiotic stresses, the five broad-spectrum protections are regulated utilized in a complicated managing network consisting of numerous mediators and gene regulatory constituents in response to abiotic stresses [132]. During the procedure, stress hormones, particularly nitrogen oxides (NO), abscisic acid (ABA), polyamines (PAs), calcium ions (Ca^2+^), hydrogen sulfide (H_2_S), reactive oxygen species (ROS), and phytochrome B (PHYB), interact with others, either synergistically or antagonistically. The transcription factors (TFs) could alter the expression of genes and enzyme activity in a regulatory way, triggering a suitable reaction. The regulatory constituents open a lot of potential for developing multiple stress tolerance/resistance. Five main plant defenses to abiotic stresses are ROS scavengers, molecular chaperones, cuticle as the outer shield, oxylipin precursors, and osmoprotectants, along with unsaturated fatty acids, and compatible solutes [132].

### 3.1. Drought Stress

Drought is becoming a challenge to sustainable agriculture due to the consequences of climate change, including erratic rainfall patterns and rising temperatures in many regions of the world. The greatest danger to global food security is drought stress, which is the primary factor in the catastrophic loss of agricultural production and productivity [133]. Drought alone can reduce yield by 50–70% in different crops [134]. For example, 40% yield losses due to drought stress have been reported in maize [35,135], 50% in rice [136], 21% in wheat [126,135], 27–40% in chickpea [125,137], 68% in cowpea [138] and 42% in soybean [34,139]. After the discovery of genome editing, efforts are being planned to alter the genes involved in pathways enabling drought tolerance, in order to increase farmers’ acceptance of crops using these technologies. In recent years, in-depth research has helped to adapt and overcome drought stress using CRISPR-Cas9 technology (Table 3).

In many crop plants, H_2_O_2_ and abscisic acid (ABA) are frequently produced in situations of salinity or drought stress. The discovery was reported of ABA-induced transcription repressors (AITRs) as a novel transcription factor family that plays a significant role as feedback regulators of ABA signaling. Alternation in the expression of AITR genes resulted in abiotic stress tolerance, including drought and salinity in *Arabidopsis* [140,141]. A CRISPR/Cas9-induced mutation in the *Arabidopsis* OST2 structural gene exhibited drought resistance [142]. Another study found that knockout of Arabidopsis plants’ genemiR169athrough CRISPR/Cas9 led to significantly improved drought tolerance [143]. Similarly, Arabidopsis’ drought tolerance increased after the vacuolar H+-pyrophosphate (AVP1) regulating gene was expressed using CRISPR/Cas9 [144]. Similar results were shown when the abscisic acid-responsive element binding gene (AREB1) was activated in Arabidopsis through CRISPR/Cas9a [145]. Recently, drought tolerance in *Arabidopsis thaliana* was demonstrated via the CRISPR/Cas9 gene silencing of the trehalose (TRE1) gene [146].

Numerous studies have documented how CRISPR confers drought resistance in many plants. For instance, it has been demonstrated that increasing rice’s ability to withstand drought can be attained by reducing the expression of the regulatory genes DERF1, PMS3, MSH1, MYB5, and SPP [147]. In rice plants, drought stress tolerance increased after OsERA1 was modified using CRISPR/Cas9 [148]. CRISPR/Cas9 has been employed to improve drought resistance in rice by knocking out the SRL1, SRL2, and ERA1 genes [148,149]. A CRISPR/Cas9-created ospyl9 mutant might increase rice yield and drought tolerance [150]. Indica mega rice cultivar MTU1010 with broader leaves, a decreased stomatal density, and improved leaf water retention under drought stress was developed using CRISPR/Cas9 to modify the *OsDST* gene [151]. The *OsOREB1*, *OsRab21*, *OsRab16b*, *OsLEA3*, *OsbZIP23*, *OsSLAC1*, and *OsSLAC7* genes, which act downstream of SAPK2, were modulated in expression in the loss-of-function sapk2 mutant of rice plants developed using CRISPR/Cas, increasing their tolerance to drought stress [131].

Two genes, *RVE7* and *4CL*, have been found to be associated with drought tolerance in chickpeas. The first report of CRISPR/Cas9-mediatedediting of the chickpea protoplast was made by Badhan et al. [152]. They described knockouts of the genes *4CL* and *RVE7*, which are linked to pathways for drought tolerance. That study established a framework for potential future chickpea-genome-editing approaches [153]. Another gene, namely *ARGOS8*, responding to drought stress has been altered through genome editing. The expression of the *ARGOS8* gene increased as a result of negative regulators of ethylene signaling pathways, providing drought tolerance [154,155]. To increase the production of maize under drought stress under field conditions, the GOS2 promoter region was replaced with an *ARGOS8* promoter sequence using the CRISPR/Cas system [156].

CRISPR/Cas9 altered the *GID1* gene in tomato plants, which exhibit high leaf water content under drought conditions [157]. Additionally, *SlLBD40* gene mutation caused by CRISPR/Cas9 significantly improved drought tolerance in tomato [158]. Furthermore, use of the CRISPR/Cas technique to alter mitogen-activated protein kinases (MAPKs) revealed SlMAPK3 to be a drought stress modulator [159]. Knockout of the *SINPR1* gene resulted in increased drought tolerance and down-regulation of drought-related genes [160].

Drought resistance of wheat was improved by CRISPR/Cas editing of wheat *TaDREB2* and *TaERF3* [89]. In wheat, a multiplex CRISPR/Cas9 assay was used to alter the *SAL1* gene, a negative regulator of drought tolerance, to increase drought tolerance at the seedling stage [161]. CRISPR/Cas genome editing of the HB12 gene can increase cotton’s resistance to drought [162]. CRISPR/Cas9 was used to modify the *BnaA6.RGA* gene in oil seed crops, which significantly improved rapeseed’s ability to withstand drought [163].

### 3.2. Heat/Temperature Stress

Plants have a preferred temperature, any rise or fall in that temperature can significantly impede their development and productivity. The third most important abiotic factor is heating stress, which may decrease crop production considerably. For instance, every 1 °C augmentation in atmospheric temperature diminishes wheat yield by 6%, rice yield by 10–20%, and corn yield by 21–31% [164,165,166]. Significant yield losses were caused by high heat stress, which is now recognized as a severe problem that will simply become worse in the future. All phases of plant growth, from germination to harvest, are severely harmed by heat stress [167,168]. Heat stress not only increases plant mortality rates but also reduces plant quality [169,170].

In severe cases, a bad alteration in temperature results in plant mortality because plants are more susceptible to temperature changes. The ideal temperature would normally be better for crop growth and development; conditions below and above the optimum temperature have a harmful effect on productivity. For every 10 °C rise, followed by 20 °C and 30 °C, mostbiochemical and enzymatic procedures double in speed [171]. Abiotic stressors, predominantly high and low heat, have a harmful effect on the premature stage of the male gametophyte in a range of agricultural crops, including maize, rice, barley, wheat, sorghum, and chickpea [172]. Due to temperature stress, the functions of tapetal cells are diminishedduring the reproductive growth period, and the anther is dysplastic. Pollen discharge is insufficient and indehiscence happens as a result of increased heat preventing pollen grains from escalating. Plants have developed precise physiological and chemical reactions to manage temperature stress [173].

The presence of genes that are responsive to heat stress, signal transduction, and the synthesis of metabolites are only a few of the complex molecular systems that plants activate in response to heat stress. Different temperature-stress-related genes have been identified and characterized to improve plants’ ability to withstand heat as a result of developments in structural and functional genomics technologies in plants. The heat stress reaction, which is connected to the accumulation of ROS, is mediated by the heat shock transcription factors (HSFs) and the heat shock proteins (HSPs) [174].Therefore, by enhancing plants’ ability to resist ROS components, temperature stress tolerance can be improved [175]. This indicated that higher tolerance might increase the antioxidant properties of crops. Plant temperature tolerance was significantly increased via metabolite production and temperature-induced gene expression. To explore the molecular processes associated with temperature stress and improve plant heat tolerance, CRISPR-Cas9 is a cutting-edge technology among all genome-editing techniques [176] (Table 3).

A cultivable HS-inducible rice mutant was created using CRISPR/Cas9 technology [177]. The orthologs of mitogen-activated protein kinase 3 and agamous-like 6 were modified using CRISPR to increase tomato sensitivity to heat stress, whereas ADP-ribosylation factor 4 enhanced tomato sensitivity to salinity shocks. According to Bouzroud et al. [178], these CRISPR-edited mutant plants had improved agronomic characteristics and were resilient to abiotic stresses. As a component for heat tolerance, BRZ1 positively regulates the formation of ROS in the tomato apoplastic area. This was confirmed by the CRISPR-Cas9-based bzr1 mutants, which showed reduced temperature tolerance and respiratory burst oxidase homolog 1 (RBOH1) with diminished hydrogen peroxide generation in the apoplast [179]. In comparison to wild-type crops, the development of CRISPR/Cas-mediated heat-stress-sensitive albino 1 (HSA1) mutants of tomato showed greater sensitivity to temperature stress [180].

The thermosensitive genic male sterile gene was altered by CRISPR in maize to promote thermo susceptible male-sterile plants [181]. In lettuce, knockouts of NCED4, a crucial regulating enzyme in abscisic acid production, allowed the seeds to germinate at a higher temperature. As a result, LsNCED4 mutants may have commercial significance in manufacturing environments with high temperatures [182]. In order to make a plant more resistant to heat, the hsps gene, which increases osmolyte levels and prevents cell protein damage, can be overexpressed [183]. The protein kinase SAPK6 and the transcription factor OsbZIP46CA1 in rice also increase the capacity for responding to heat stress [184].

### 3.3. Cold Stress

Cold stress, which includes chilling (20 °C) and freezing (0 °C) temperatures, hinders plant growth and development and severely limits plant geographic expansion and agricultural productivity [185]. Plants are directly inhibited from responding metabolically to low temperatures, which results in osmotic stress, oxidative stress, and other types of stress. Due to mechanical damage and metabolic dysfunction caused by extreme cold temperatures, plant growth and development are halted [186]. The physiological, biochemical, and molecular behavior of plants during their growth and expansion is adversely affected by cold stressors. The photosynthetic capacity and crop anatomy are brutally impacted by cold exposure, especially throughout the winter [187,188].Cold stress during the seedling stage may cause impaired germination and emergence. Long-term exposure impairs source–sink relationships, growth, nutrient localization, and leaf chlorosis [189]. Membrane formation, which amplifies other cold-stress-related downstream processes, is the main consequence of cold stress on crops [190]. In-generic or inter-specific hybridization has been successful in boosting the cold tolerance of significant crops using conventional breeding methods. For creating non-transgenic genome-edited crops to combat climate change and ensure future food security, CRISPR/Cas9 is a clever and practical approach [191,192] (Table 4).

To increase the plant’s resistance to cold, genome editing is employed to target a few of the depressant regulator transcription factors in rice. A transcription factor called OsMYB30 attaches to the amylase gene promoter and negatively affects cold tolerance. According to Lv et al. [193], under conditions of cold stress, OsMYB30 forms a compound with OsJAZ9 and slows down the expression of the amylase gene, which may contribute to increasing cold sensitivity by causing maltose buildup and starch breakdown. In order to determine the specific function of the TIFY1a, TIFY1b, and Ann3 genes in rice’s ability to withstand cold stress, CRISPR/Cas9 technology has also been applied to these genes. The mutant outperformed the natural variation in terms of yield, temperature tolerance, and amount of germination prior to harvest [194]. Using CRISPR base editing, suppression of photosynthetic genes in rice plants under cold stress has been shown to cause the white-striped leaves phenotype in the white stripe leaf 5 (wsl5) mutant line [195,196].

PRPs are proline-rich proteins that not only aid in dealing with low temperatures but also reduce nutrient loss, boost antioxidant activity, and aid in the production of chlorophyll. Rice capacity for cold tolerance was improved by the CRISPR/Cas9 deletion of OsPRP1, which encodes a proline-rich protein [197]. In a recent work using CRISPR/Cas9, three rice genes, viz., OsPIN5b, GS3, and OsMYB30were altered to increase spike length, grain size, and resilience to cold stress [198]. The CRISPR/Cas9 technology altered the G-complex-related genes i.e., OsRGA1, OsGS3, OsDEP1, and OsPXLG4 to make rice more resistant to chilling stress [199].Because tomato plants are prone to chilling stress, their fruits are more vulnerable to damage from the cold. C-repeat binding factor 1 (CBF1) was shown using CRISPR-Cas9-based cbf1 mutants to protect the tomato plant next to it from cold/chilling damage and decrease electrolyte leakage [200]. These plants also demonstrated excellent addition of hydrogen peroxide and indole acetic acid, resulting in tomato plants tolerant of chilling stress.

**Table 3:** List of reported targeted gene(s) via CRISPR/Cas9 technology in different plant species for development of tolerant genotypes against drought and heat stresses.

Crops	Gene	Trait	Technique	References
Rice	*OsDERF1*	Drought	CRISPR/Cas9	[147]
Rice	*SRL1*, *SRL2*	Drought	CRISPR/Cas9	[149]
Rice	*OsAAA-1*, *OsAAA-2*	Drought	CRISPR/Cas9	[201]
Rice	*OsNAC006* (transcription factor)	Drought and heat sensitivity	CRISPR/Cas9	[202]
Rice	*OsAOX1a*	Drought resistance	CRISPR/Cas9	[147]
Rice	*OsDST*	Drought and salinity	CRISPR/Cas9	[151]
Rice	*OsERA1, OsPYL9*	Drought	CRISPR/Cas9	[148,150]
Rice	*SAPK2*	Tolerance to salinity and drought	CRISPR/Cas9	[131]
Rice	*OsPMS3*	Photoperiod-sensitive male-sterile	CRISPR/Cas9	[147]
Rice	*Csa*	Photosensitive-genic male-sterile	CRISPR/Cas9	[203,204]
Rice	*TMS5*	Thermo-sensitive genic male-sterile	CRISPR/Cas9	[205]
Rice	*OsNAC14*	Drought tolerance	CRISPR/Cas9	[206]
Rice	*OsPUB67*	Drought tolerance	CRISPR/Cas9	[207]
Wheat	*TaDREB2, TaERF3*	Tolerance to drought	CRISPR/Cas9	[89]
Maize	*ZmARGOS8*	Drought	CRISPR/Cas9	[156]
Maize	*ZmTMS5*	Creation of thermosensitive maize lines	CRISPR/Cas9	[181]
Mustard	*BnaA6.RGA*	Drought tolerance	CRISPR/Cas9	[163]
Soybean	*Drb2a, Drb2b*	Tolerance to drought and salinity stress	CRISPR/Cas9	[208]
Soybean	*GmMYB118*	Drought tolerance	CRISPR/Cas9	[209]
Chickpea	*4CL, RVE7*	Drought tolerance	CRISPR/Cas9	[152]
Tomato	*SIMAPK3* and *SlNPR1*	Drought	CRISPR/Cas9	[159,160]
Tomato	*SlARF4*	Drought	CRISPR/Cas9	[140]
Tomato	*SIAGL6*	Heat stress	CRISPR/Cas9	[210]

**Table 4:** List of reported targeted gene(s) via CRISPR/Cas9 technology in different plant species for development of tolerant genotypes against cold stresses.

Crops	Gene	Trait	Technique	References
Rice	*OsMYB30*	Cold tolerance	CRISPR/Cas9	[198]
Rice	*OsAnn3*	Cold tolerance	CRISPR/Cas9	[211]
Rice	*OsAnn5*	Cold tolerance	CRISPR/Cas9	[211]
Rice	*OsPRP1*	Cold tolerance	CRISPR/Cas9	[212]
Tomato	*SlCBF1*	Cold tolerance	CRISPR/Cas9	[200]
*Arabidopsis thaliana*	*AtCBF1*, *AtCBF2*	Cold tolerance	CRISPR/Cas9	[213]

### 3.4. Salinity Stress

Owing to the negative consequences of climate change, salinity stress has recently become much worse [214]. Salinity stress is the second most severe abiotic danger that affects fertile lands as well as crop productivity [215]. According to Morton et al. [216] and Van Zelm et al. [217], severe salts have an impact on about one-fifth of the irrigated agricultural area. Lack of good irrigation water, a changing climate, and excessive use of chemicals such as fertilizers and pesticides prolong the process of adding more land to the salinity stress zone. According to estimates made by Jamil et al. [218], 50% of cultivable lands will be saline by 2050 due to the overuse of chemicals including fertilizers and pesticides. One of the most important and harmful factors that has a negative impact on soil quality and agricultural output is salt stress. When too many soluble salts accumulate in the crop root zone, it causes salinization of the soil because roots are unable to absorb water. Thus, osmotic stress and nutritional imbalance in plants have a negative impact on their morphology, biochemistry, and biomass, which ultimately causes irreparable plant damage [219,220,221].

Reactive oxygen species (ROS) are intensified by salt stress, which has a detrimental effect on crops’ cellular and metabolic processes [222,223]. Lipid peroxidation, which causes membrane deterioration as well as protein and DNA damage, is a harmful effect of ROS [224]. By diminishing chlorophyll content and stomatal conductance, salt stress hinders the development of the photosystem II and the transpiratory apparatus [225]. Additionally, it decreases the water potential of the soil and leaves, which lowers plant turgor pressure by affecting water relations and causing osmotic stress [226]. Plants suffer from decreased leaf area, lower photosynthetic rate, poor seed germination, decreased biomass production, and crop yield as a result [227,228,229]. Salinity tolerance is the ability of a plant to maintain the equilibrium of biomass and/or output under conditions of salt stress. In order to tolerate salt, plants have several molecular and physiological mechanisms [230].

Genome editing has the capacity to improve crops; there are yet few studies on its effective application in breeding plants that can withstand saline stress (Table 5). In one such work, rice was modified to impart salt stress tolerance by editing the *OsRR22* gene, which encodes for a transcription factor (TF) involved in the control of signaling and the metabolism of cytokinins in plants [231,232]. Using CRISPR/Cas9 technology, the *OsRR22* gene was altered, and two homologous T_2_ generations revealed improved salt tolerance with no discernible difference between the modified and wild-type lines [232]. Using CRISPR/Cas9 technology, the paraquat tolerance-3 mutations (*OsPQT3*) gave rice a high level of salt tolerance [233]. The function of *OsmiR535* in salt stress tolerance was investigated using genome-editing techniques, and it was proposed that *OsmiR535* might be knocked out using CRISPR/Cas9 to enhance salinity tolerance in rice. Additionally, a homozygous 5bp deletion in the *OsmiR535* coding region might be a valid target for raising rice’s salt tolerance [234]. Furthermore, some other genes increase the ability of rice to tolerate salt, using CRISPR/Cas9 technology by eliminating the *OsbHLH024* gene and increasing the expression of the ion transporter genes including *OsHKT1*;3, *OsHAK7*, and *OsSOS1* [235]. When the rice *OsRAV2* gene was altered using CRISPR-Cas, the rice plants were able to survive under high salt conditions [236].

Improvements in salt stress tolerance were seen in tomatoes after changes were made to the 8CM and PRD domains of the hybrid proline-rich protein1 (HyPRP1) [247]. Additionally, the capability of crops to tolerate salt stress may be significantly increased by employing CRISPR/Cas9 technology to eliminate the *OsDST* genes for rice [151], *OsNAC041* [238], and HvITPK1 [246] for barley.

### 3.5. Heavy Metals Stress

An important issue for sustainable agricultural development is heavy metals, which seriously impair plant growth and productivity [249]. Heavy metals (HMs) including Mn, Cu, Ni, Co, Cd, Fe, Zn, and Hg, among others, have accumulated in soils as a result of various human activities such the application of fertilizer, incorrect disposal of industrial waste, and unauthorized sewage disposal [250,251], or the hasty disposal of vehicle waste. They are either collected on the soil surface or leached from the soil into the groundwater [252,253]. Additionally, heavy metals cause oxidative stress by promoting the generation of hydroxyl radicals (OH), superoxide radicals, and hydrogen peroxide (H_2_O_2_) [250,254]. Plant physio-morphological activities are hampered by the accumulation of HMs, especially in the roots where they are blocked by Casparian strips or trapped by root cell walls, which eventually reduces crop output [255]. When consumed, heavy metals accumulated in plants canseriously impair human health [256].

To combat heavy metal stress in plants, CRISPR-Cas9-induced plant mutants may prove useful (Table 6). In contrast to WT Co10 plants, the oxp1/CRISPR mutant of Arabidopsis plants exhibits resistance to Cd, indicating an increased capacity for heavy metal detoxification in mutant crops [257]. Accordingly, study showed how indel mutations using gene-editing techniques could provide tolerance to heavy metals and xenobiotics in plants [257]. Increased plant tolerance to heavy metals is influenced by a variety of genes [258]. Several transporter genes in rice, including OsLCT1 and OsNramp5, are implicated in Cd absorption by the roots [259]. The amount of Cd in rice has been reduced by CRISPR/Cas9-enabled gene-expression manipulation. Rice grains with OsNRAMP1 knocked out by CRISPR/Cas9 have decreased levels of Cd and lead (Pb) [260,261]. Eliminating an R2R3 MYB transcription factor called OsARM1 using CRISPR/Cas9 prevents rice from absorbing and transporting arsenic [262].Cesium (Cs+) absorption and translocation in rice are regulated by the *OsHAK1* gene. Using the CRISPR-Cas9 technique, the cesium permeable potassium transporter *OsHAK1* was turned inactive [263].

### 3.6. Herbicide Stress

In order to increase crop productivity, there is a need to manage weed growth with application of herbicides. Herbicides destroy non-target plants while also causing stress to the target plants and weed plants by interfering with or changing their metabolic processes. They also leave soil residues that are hazardous to the environment [264,265].The morphological, physiological, and biochemical traits of agricultural plants have been negatively impacted by the inappropriate application of herbicides. Herbicide toxicity reduces photosynthetic activity, which has a detrimental impact on the ability of crop plants to produce yield. One of the main goals for raising agricultural productivity is the development of herbicide tolerance in crop plants. To improve herbicide resistance in plants, genome editing including ZFNs, TALENs, and CRISPR/Cas technologies is an excellent tool (Table 6).

Leucine, isoleucine, and valine are branched amino acids whose biosynthesis is catalyzed by the enzyme acetolactate synthase, which is encoded by the *ACETOLACTATE SYNTHASE* (*ALS*) gene [266,267]. It is a potential target of many herbicide improvement programmes. The recombination of acetolactate synthase using CRISPR/Cas9 produces herbicide resistance in rice [268] and in watermelons [269]. Additionally, using the same strategy and emphasizing the *ALS1* and *ALS2* genes, herbicide-resistant maize plants were produced [270]. CRISPR-based editing in the *OsALS1* gene has been used to introduce herbicide tolerance characteristics into rice [271,272]. Glyphosate is one of the most imperative and quickly adopted herbicides for function in resistant crops such as soybean, maize, sugar beet, and chili pepper. The advancement of glyphosate-resistant plants requires changes in the machinery of some genes [203]. 5-enolpyruvylshikimate-3-phosphate synthase (EPSPS) enzyme is implicated in the formation of aromatic compounds in crops with the transfer of phosphoenolpyruvate (PEP) enzyme for activating the reaction [203,273]. Glyphosate hinders the act of the EPSPS enzyme by inhibiting the add-on of glyphosate to the PEP enzyme binding sites, eventually blocking the formation of aromatic products and causing crop death [203]. The endogenous EPSPS gene of rice was targeted with CRISPR/Cas9 to produce site-specific gene incorporation and substitution, which were fully transferred to the next generation with crops 100% resistant to the glyphosate [203]. CRISPR/Cas9 was also utilized toproduce a mutation in the promoter of the EPSPS gene of chili to state this gene beneath the action of glyphosate [274]. The resulting crops were reasonably resistant to glyphosate, and additional studies advised that selecting a diverse promoter may assist in the development of entirely resistant chili [274].The modified genotypes of rice and flax now have enhanced tolerance to glyphosate as a result of the CRISPR/Cas9 change of two nucleic acid residues in the binding site of glyphosate–EPSPS [91,203]. Recently, herbicide resistance was developed in tomato plants by CRISPR-Cas9-based targeted mutations in EPSPS, PDS (phytoene desaturase), and ALS [92].

**Table 6:** List of reported targeted gene(s) via CRISPR/Cas9 technology in different plant species for tailoring herbicide and metal stress tolerance.

Crops	Gene	Trait	Technique	References
Rice	*C287T*	Herbicide resistance	CRISPR/Cas9	[274]
Rice	*BEL*	Herbicide resistance	CRISPR/Cas9	[71]
Rice	*OsALS1*	Herbicide tolerance	CRISPR/Cas9	[271]
Rice	*EPSPS*	Herbicide resistance	CRISPR/Cas9	[203]
Rice	*SF3B1*	Herbicide resistance	CRISPR/Cas9	[72]
Wheat	*ALS*	Herbicide resistance	CRISPR/Cas9	[275,276]
Maize	*ALS1 and ALS2*	Herbicide resistance	CRISPR/Cas9	[270]
Maize	*MS26*	Herbicide resistance	CRISPR/Cas9	[270]
Soybean	*ALS1*	Resistant to Chlorsulfuron	CRISPR/Cas9	[277]
Tomato	*ALS*	Resistant to Chlorsulfuron	CRISPR/Cas9	[278]
Tomato	*SlEPSPS*	Herbicide resistance	CRISPR/Cas9	[92]
Tomato	*SlALS1*, *SlALS2*	Herbicide resistance	CRISPR/Cas9	[92]
Tomato	*Slpds1*	Herbicide resistance	CRISPR/Cas9	[92]
Rice	*OsTubA2*	Base editing	CRISPR/Cas9	[279]
Rice	*OsHAK1*	Low cesium accumulation	CRISPR/Cas9	[263]
Rice	*OsPRX2*	Potassium deficiency tolerance	CRISPR/Cas9	[280]
Rice	*OsARM1*	Increase tolerance to higharsenic	CRISPR/Cas9	[260]
Rice	*OsLCT1*	Less cadmium accumulation	CRISPR/Cas9	[259]

## 4. Conclusions and Prospects

Plants serve as sources of food, fiber, medicine, biofuels, and other goods. Farmers need new, superior cultivars in order to increase crop output and feed both the nation and the world. Plant breeders need a variety of tools for this purpose, including genomics and marker-assisted molecular breeding. Scientists can now implant desired traits more precisely and faster than in the past. Meganucleases (MNs), zinc finger nucleases (ZFNs), transcription activator-like effector nucleases (TALENs), and the clustered regularly interspaced short palindromic repeats (CRISPR) system are genome-editing tools that have been used with greater accuracy and efficiency than conventional breeding to enhance the quality of staple, oilseed, and horticultural crops. Today, there are several successful cases of “genome editing.” In order to edit genes accurately in the genomes of model and crop plants as well as a range of other organisms, genome editing employs designed nucleases as potent tools that target certain DNA sequences. A study of the literature on transcriptomics, biotechnology, genomics, and phonemics has shown that this novel approach to crop development is effective. CRISPR/Cas9-based genome editing is a genuinely innovative strategy. With genome editing, crops can effectively incorporate a variety of genetic traits. When these precise and powerful methods are applied to expedite plant breeding, they create certain outcomes. In order to accomplish a second Green Revolution and meet the escalating food demands of a quickly growing global population under constantly changing climatic conditions, plant breeding will advance with the help of this multidisciplinary approach. By overcoming the limitations of current transgenic techniques, genome-editing technology ushers in a new era of improved plant genetics. This information may be proved useful to plant breeders and researchers in their thorough evaluation of the use of various gene-editing tools to improve crops by focusing on the targeted gene. We believe that CRISPR/Cas9 technology islikely to bridge the GMO and societal divide in upcoming days.

## Figures and Tables

**Table 5:** List of reported targeted gene(s) via CRISPR/Cas9 technology in different plant species for developing salinity tolerance.

Crops	Gene	Trait	Technique	References
Rice	*OsbHLH024*	Salinity	CRISPR/Cas9	[235]
Rice	*OsRR22*	Salinity	CRISPR/Cas9	[232,237]
Rice	*OsRAV2*, *OsNAC041*, *OsmiR535*	Salinity	CRISPR/Cas9	[234,236,238]
Rice	*OsRR9*, *OsRR10*	Salinity	CRISPR/Cas9	[239]
Rice	*OsNAC041*	Salinity	CRISPR/Cas9	[240]
Rice	*OsOTS1*	Salinity	CRISPR/Cas9	[241,242]
Rice	*OsDST*	Drought and salinity	CRISPR/Cas9	[151]
Rice	*SAPK2*	Tolerance to salinity	CRISPR/Cas9	[131]
Wheat	*TaHAG1*	Salt tolerance	CRISPR/Cas9	[243]
Maize	*ZmHKTI*	Tolerance to salinity	CRISPR/Cas9	[244]
Soybean	*GmAITR*	Salt tolerance	CRISPR/Cas9	[245]
Soybean	*Drb2a*, *Drb2b*	Tolerance to droughtand salinity stress	CRISPR/Cas9	[208]
Barley	*HvITPK1*	salinity	CRISPR/Cas9	[246]
Tomato	*SlHyPRP1*, *SlARF4*	salinity	CRISPR/Cas9	[247,248]
